# Pharmacological AMP Kinase Activators Target the Nucleolar Organization and Control Cell Proliferation

**DOI:** 10.1371/journal.pone.0088087

**Published:** 2014-01-30

**Authors:** Mohamed Kodiha, Ali Salimi, Yi Meng Wang, Ursula Stochaj

**Affiliations:** Department of Physiology, McGill University, Montreal, Quebec, Canada; George Mason University, United States of America

## Abstract

**Aims:**

Phenformin, resveratrol and AICAR stimulate the energy sensor 5′-AMP activated kinase (AMPK) and inhibit the first step of ribosome biogenesis, *de novo* RNA synthesis in nucleoli. Nucleolar activities are relevant to human health, because ribosome production is crucial to the development of diabetic complications. Although the function of nucleoli relies on their organization, the impact of AMPK activators on nucleolar structures is not known. Here, we addressed this question by examining four nucleolar proteins that are essential for ribosome biogenesis.

**Methods:**

Kidney cells were selected as model system, because diabetic nephropathy is one of the complications associated with diabetes mellitus. To determine the impact of pharmacological agents on nucleoli, we focused on the subcellular and subnuclear distribution of B23/nucleophosmin, fibrillarin, nucleolin and RPA194. This was achieved by quantitative confocal microscopy at the single-cell level in combination with cell fractionation and quantitative Western blotting.

**Results:**

AMPK activators induced the re-organization of nucleoli, which was accompanied by changes in cell proliferation. Among the compounds tested, phenformin and resveratrol had the most pronounced impact on nucleolar organization. For B23, fibrillarin, nucleolin and RPA194, both agents (i) altered the nucleocytoplasmic distribution and nucleolar association and (ii) reduced significantly the retention in the nucleus. (iii) Phenformin and resveratrol also increased significantly the total concentration of B23 and nucleolin.

**Conclusions:**

AMPK activators have unique effects on the subcellular localization, nuclear retention and abundance of nucleolar proteins. We propose that the combination of these events inhibits *de novo* ribosomal RNA synthesis and modulates cell proliferation. Our studies identified nucleolin as a target that is especially sensitive to pharmacological AMPK activators. Because of its response to pharmacological agents, nucleolin represents a potential biomarker for the development of drugs that diminish diabetic renal hypertrophy.

## Introduction

5′-AMP activated kinase (AMPK) serves as an energy sensor that is implicated in numerous biological processes. As a ser/thr protein kinase, AMPK provides a focal point for metabolic control in all eukaryotes, where it exerts essential functions in different organs and cell types [1], [2], [3], [4], [5]. Owing to its critical role in glucose, lipid and protein homeostasis, AMPK is crucial for many human diseases and disorders and has become an important therapeutic target for type 2 diabetes and obesity ([2], [3], [5], [6] and references therein). The kidney is one of the organs affected by diabetic complications [7], [8], [9], [10], [11], [12]; the proximal tubule in particular displays hyperplasia followed by hypertrophy at the early stages of diabetes [13]. We have previously used cells of the proximal tubule to investigate the role of AMPK in cell physiology [14], while other studies in kidney cells demonstrated the importance of AMPK for protein translation [15]. Furthermore, on the organismal level, the link between AMPK and kidney disease is well established [7], [16], [17]. Thus, it was proposed that the drop in AMPK activity following hyperglycemia upregulates protein synthesis in the kidney and ultimately leads to renal hypertrophy [7], [16], [18]. The cause-effect relationship between AMPK and renal hypertrophy was revealed with the AMPK activator resveratrol (*trans*-3,4′,5-trihydroxystilbene); this compound prevented the hyperglycemia-induced upregulation of protein translation [7]. However, the impact of pharmacological agents on protein synthesis is likely more complex, because AMPK activators interfere with the first step of ribosome biogenesis, *de novo* RNA synthesis in the nucleolus [14]. Since there is only limited information available on how AMPK activators affect the nucleolus, it was our goal to address this question at the cellular and subcellular level.

The nucleolus is a specialized compartment in the nucleus that has emerged as a key player for many aspects of cell biology. Nucleoli transcribe ribosomal RNA, assemble ribosomal subunits and signal recognition particle (SRP), control apoptosis, cell cycle progression, p53, telomerase, stress responses and virus replication [19], [20], [21], [22], [23], [24]. The nucleolus is organized into subcompartments that differ in their biological functions. Within the tripartite nucleolus of mammalian cells, fibrillar centers (FC) and dense fibrillar components (DFC) are embedded in the granular component (GC). With up to several thousand different proteins [25], [26], the organization and composition of nucleoli is not static, but modulated by disease, stress and environmental changes [20], [27], [28]. In particular, nucleophosmin/B23 (here referred to as B23), fibrillarin, nucleolin and RPA194 are dynamic and essential components of the nucleolus which can serve as marker proteins to monitor changes in nucleolar organization ([14], [29]; Su et al., unpublished).

Several lines of evidence link nucleolar proteins to insulin-depending signaling or diabetes. For example, nucleolin and B23 are phosphorylated in response to insulin treatment [30], [31]. On the other hand, high glucose concentration promotes the association between upstream binding factor UBF and the largest RNA polymerase I subunit RPA194 in glomerular epithelial cells. This interaction is believed to promote rDNA transcription and thereby ribosome biogenesis [32]. Aside from biochemical data, genetic studies implicate the nucleolar protein encoded by *C2orf37* in diabetes [33]. Moreover, proteomics detected a fragment of insulin receptor substrate 2 in nucleoli [34].

At the functional level, both the localization and concentration of B23, fibrillarin, nucleolin and RNA polymerase I subunits in nucleoli are crucial for rDNA transcription, pre-rRNA processing and ribosome biogenesis [20], [21], [22], [23], [27], [35], [36], [37], [38], [39]. Within the nucleolus these proteins are concentrated in different subcompartments; B23 resides in the GC, fibrillarin in the DFC, RPA194 in the FC, and nucleolin has been detected in both the DFC and GC ([39] and references therein).

We have demonstrated previously that pharmacological AMPK activators reduce significantly *de novo* RNA synthesis in nucleoli [14]. Specifically, phenformin, resveratrol and AICAR (5-Aminoimidazole-4-carboxyamide ribonucleoside) inhibit transcription in the nucleolus, and the most pronounced effect is observed with resveratrol. To date, the mechanisms underlying the drug-induced changes in nucleolar function are not known. However, this knowledge is important, because the compounds tested by us are directly relevant to the treatment of diabetes. A better understanding of the drug targets and their physiological effects may open new avenues to improve therapeutic intervention for obesity, metabolic syndrome, type 2 diabetes and the complications associated with these conditions.

The functioning of nucleoli relies on their proper organization [24]. Therefore, we hypothesized that pharmacological agents that activate AMPK trigger a re-organization of nucleoli. To test this model, our studies focused on four proteins that essential for ribosome biogenesis and reside in different nucleolar subcompartments.

## Materials and Methods

### Cell Culture and Incubation with Pharmacological Compounds

LLC-PK1 cells are kidney proximal tubule epithelial cells [40]; they were cultured as described [14]. Appropriate concentrations of phenformin, resveratrol or AICAR (5-aminoimidazole-4-carboxyamide ribonucleoside) and their effects on AMPK have been determined previously [14]. In brief, cells were treated with 5 mM phenformin, 200 µM resveratrol or 1 mM AICAR for 1 h at 37°C. Phenformin and resveratrol were dissolved in DMSO; the solvent was present during the incubation at a final concentration of 0.4%. AICAR was added as an aqueous solution. To induce apoptosis, LLC-PK1 cells were incubated for 24 h in medium containing 1 µM staurosporine [41]. In control experiments, cells were incubated at 37°C with 10 µg/ml cycloheximide for 1 h [42].

### DNA Synthesis

DNA synthesis was measured with click-technology. To this end, newly synthesized DNA was labeled with 5-ethynyl-2′-deoxyuridine (EdU) in combination with Alexa Fluor555 azide according to the manufacturer (Molecular Probes). In brief, cells were incubated with vehicle or AMPK activator in the presence of 10 µM EdU for 1 h at 37°C. Samples were then fixed and processed for the detection of EdU; nuclei were stained with 4′,6-diamidino-2-phenylindole (DAPI).

### Immunofluorescent Staining and Quantitative Confocal Microscopy

Immunolocalization of proteins and quantification of nucleolar, nuclear and cytoplasmic fluorescence followed published procedures [14], [43], [44]. Immunostaining was performed with antibodies against B23 (Cell Signaling; #3542; diluted 1∶700), fibrillarin (Santa Cruz, sc-25397; 1∶500), nucleolin (sc-13057; 1∶1,000), RPA194 (sc-48385; 1∶200) or lamin A (sc-20680; 1∶500). Using MetaXpress software modules, nucleoli were identified with the Detect light holes filter for B23, fibrillarin or nucleolin, and the Detect dark holes filter for RPA194. The staining pattern obtained for DAPI provided the reference for dark holes. To evaluate *de novo* DNA synthesis in nuclei, EdU was labeled with Alexa Fluor555 azide and images were acquired with a Zeiss LSM510 confocal microscope, using a 20×objective (NA = 0.5) and a zoom of 2 as described [45]. Pixel intensities were measured for all nuclei, which were demarcated by DAPI staining.

### Whole Cell Extracts, Cell Fractionation and Quantitative Western Blotting

Procedures for the preparation of crude extracts, nuclear or cytoplasmic fractions and quantitative Western blotting have been described in detail [14], [46]. The cell fractionation protocol includes a wash step with 0.005% Nonidet P-40. Under these conditions, molecules that are not tightly associated with nuclei will be recovered in the cytoplasmic fraction. To assess apoptosis, cells attached and floating in the medium were collected and combined for Western blot analysis. Primary antibodies were used for blotting at the same concentration as for immunostaining. In addition, antibodies against nucleolin (sc-55486; 1∶500), phospho(Ser10)-histone H3 (Cell Signaling, #3377; 1∶1,000), cleaved lamin A (Cell Signaling, #2031; 1∶500); lactate dehydrogenase (Rockland; 1∶4,000), PARP1 (sc-25780; 1∶1,000) or actin (Chemicon; 1∶100,000) were diluted as indicated.

### Statistics

**Figure 1:**
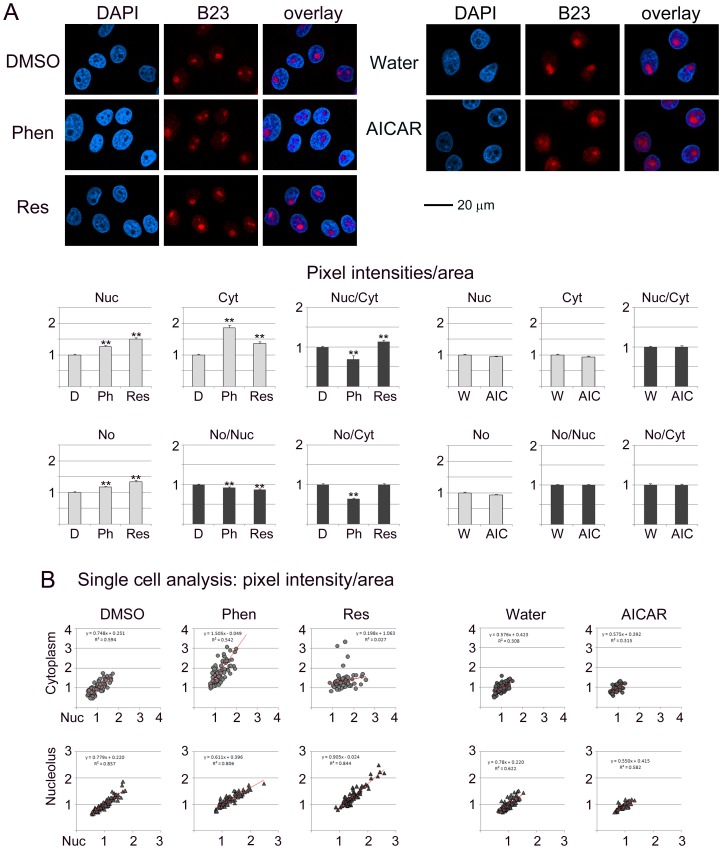
The pharmacological compounds phenformin, resveratrol and AICAR alter the compartment-specific concentration and subcellular distribution of the nucleolar protein B23. B23 was detected by indirect immunolocalization in controls treated with vehicle (DMSO, D; water, W), phenformin (Phen, Ph), resveratrol (Res) or AICAR (AIC). Confocal images were acquired and fluorescence intensities were quantified for subcellular compartments. (A) Antibody staining is shown for B23, nuclei were identified with DAPI (4′,6-diamidino-2-phenylindole). Size bar is 20 µm. Graphs depict the changes in nuclear (Nuc), cytoplasmic (Cyt) and nucleolar (No) compartments. Data are shown as pixel intensities/area+SEM. Fluorescence intensities for controls (DMSO, Water) were defined as 1. Furthermore, the nuclear/cytoplasmic, nucleolar/nuclear and nucleolar/cytoplasmic ratio was calculated for each treatment. (B) Single cell analyses were performed to evaluate changes in the nuclear/cytoplasmic and nucleolar/nuclear distribution of B23. Trendlines were added and results for linear regression are shown.

**Figure 2:**
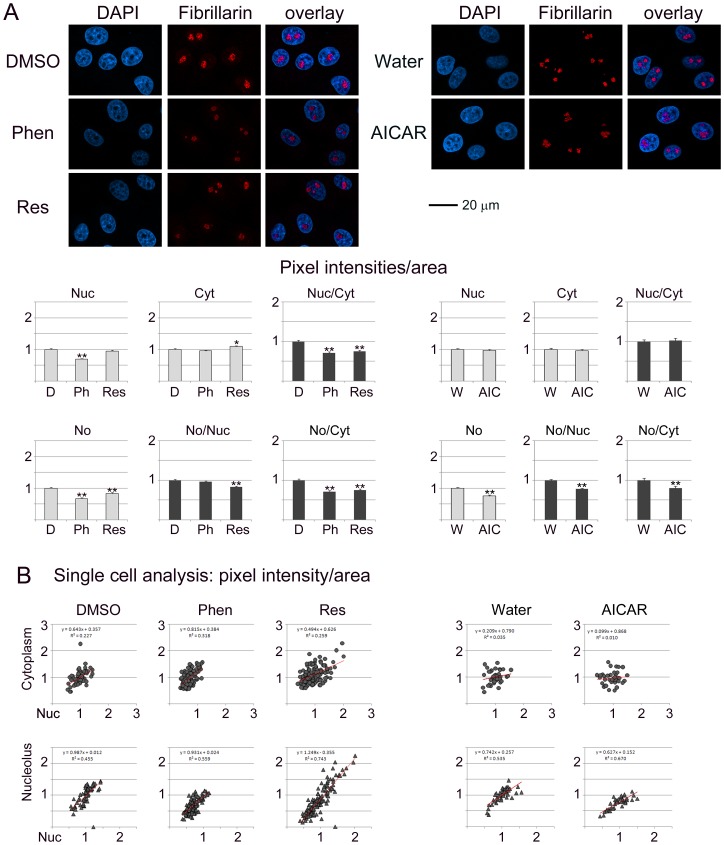
Impact of phenformin, resveratrol and AICAR on fibrillarin. Experiments were performed and data analyzed as described in detail for Fig. 1.

**Figure 3:**
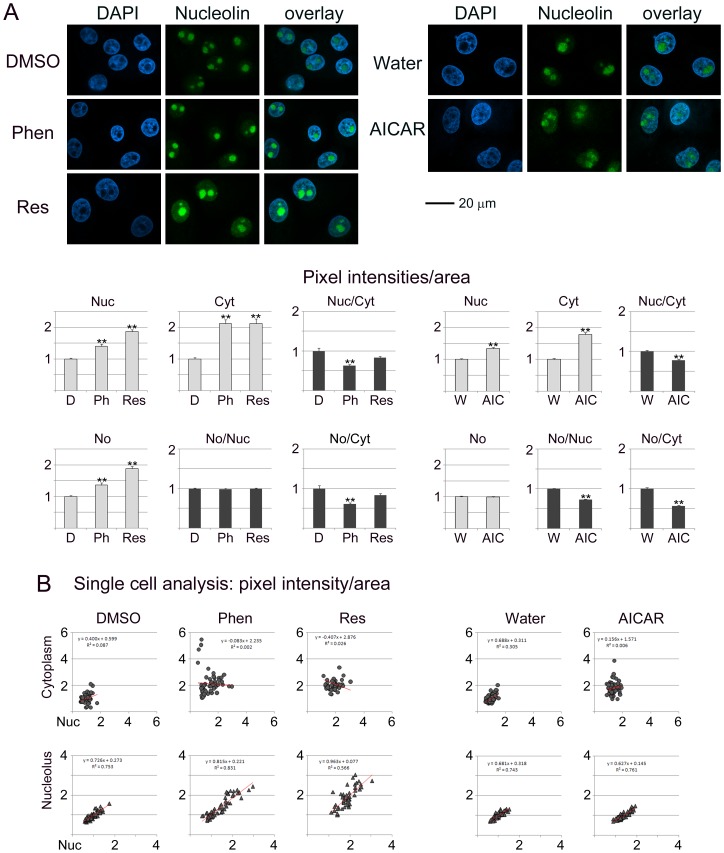
Phenformin, resveratrol and AICAR target nucleolin. Experimental design and data analysis followed the methods depicted for Fig. 1.

**Figure 4:**
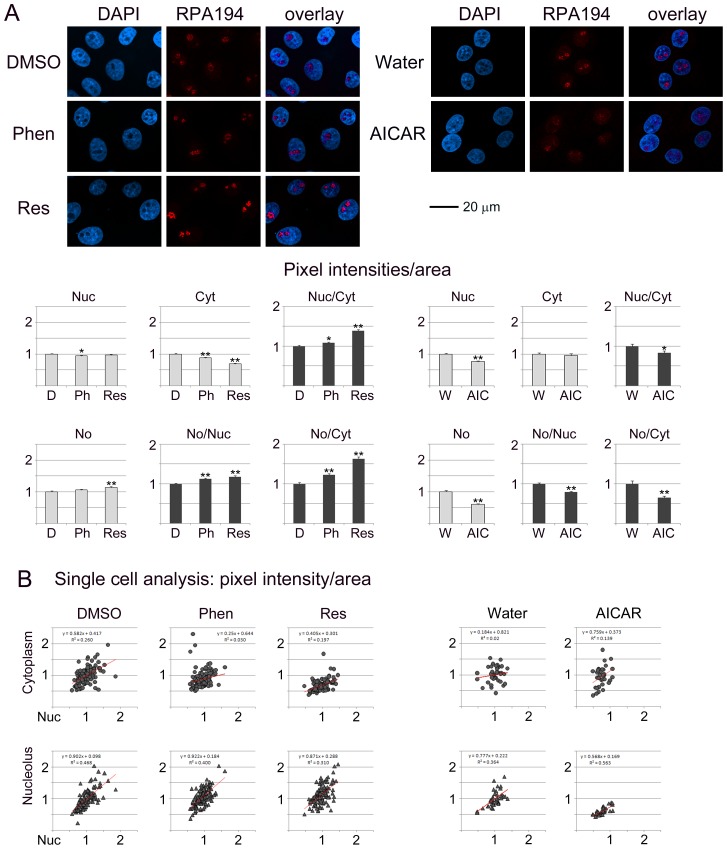
RPA194 subcellular abundance and distribution are modulated by phenformin, resveratrol and AICAR. A detailed description of the experimental set-up and data analyses is provided for Fig. 1.

Comparisons between control and treated groups were performed with Student’s t-test; data are depicted as means+SEM. Differences were considered significant if *P* values were smaller than 0.05; _**_denotes *p*<0.01; _*_indicates *p*<0.05. To quantify possible changes in nucleolar organization, at least 27 cells were investigated for each data point shown in Fig. 1–4, part A. A minimum of 50 cells was measured for each data point in Fig. S1. At least three independent sets of experiments were quantified for cell fractionation, the measurements of protein abundance or EdU incorporation.

## Results

### Rationale for Analyzing the Impact of AMPK Activators on Nucleolar Organization

Our previous studies demonstrated that pharmacological AMPK activation causes a significant reduction in *de novo* RNA synthesis in nucleoli [14]. Others have shown that the biological functions of the nucleolus are connected to the organization of this nuclear compartment (reviewed in [24]). To obtain information on the possible link between drug-induced changes in rDNA transcription and nucleolar organization, we examined B23, fibrillarin, nucleolin and RPA194. We selected these proteins for two reasons; first, they are essential for nucleolar functions, as they participate in pre-rRNA transcription and/or subsequent steps of ribosomal biogenesis. Second, they are concentrated in distinct parts of the nucleolus, and therefore provide information on different subcompartments.

Many nucleolar proteins are mobile and can be found in multiple subcellular locations. These proteins may shuttle between the cytoplasm and the nucleus. Moreover, inside the nucleus they are not restricted to nucleoli, but also reside in the surrounding nucleoplasm. We and others have previously shown that B23 and fibrillarin are highly dynamic, and their distribution is sensitive to stress ([29] and references therein). To measure such changes in subcellular localization, our group developed reliable imaging and image-analysis tools. The methods quantify fluorescence intensities in the nucleus, cytoplasm and nucleoli and provide in-depth information at the single cell level. This is important, because the protocols uncover whether a cell population responds to treatment in a uniform or heterogeneous fashion [29]. We have now applied these tools to measure the impact of phenformin, resveratrol and AICAR on four crucial proteins of the nucleolus.

### Pharmacological AMPK Activators Change the Subcellular Localization of B23, Fibrillarin, Nucleolin and RPA194

Figures 1–4 analyze the impact of phenformin, resveratrol and AICAR on the subcellular distribution of B23, fibrillarin, nucleolin and RPA194. To achieve this, protein abundance was quantified in the nucleus (Nuc), cytoplasm (Cyt) and nucleolus (No). To simplify the comparison between different compartments, results were normalized to control samples (vehicle) for each treatment.

Nucleolar proteins can regulate cell functions through their abundance in a specific location. At the same time, the balance between different compartments can also determine the ultimate response to pharmacological intervention. To address this point, the ratio for nucleolar/nuclear and nucleolar/cytoplasmic signals was calculated for each protein (part A of Fig. 1–4). Single cell data (part B of Fig. 1–4) revealed whether the response to treatment was variable within the cell population. We were particularly interested in single cell data for the nucleolar/nuclear and nucleolar/cytoplasmic distribution, because nucleolar proteins move between these compartments.

### B23 is a Target for Phenformin and Resveratrol

Fig. 1A suggests that the subcellular distribution of B23 was sensitive to the treatment with phenformin and resveratrol, whereas AICAR had little or no effect on the parameters measured. Specifically, phenformin and resveratrol increased significantly the abundance of B23 in the nucleus, cytoplasm and nucleolus. However, the increase did not occur uniformly in all subcellular compartments; consequently, the nuclear/cytoplasmic, nucleolar/nuclear and nucleolar/cytoplasmic ratios were affected.

Single cell analyses (Fig. 1B) revealed that under control conditions (DMSO or water) the distribution of nuclear/cytoplasmic B23 or nucleolar/nuclear B23 fits a linear relationship. Although this applied to treated cells as well, the slope changed with phenformin and resveratrol.

Taken together, phenformin and resveratrol altered (a) the balance between nuclear and cytoplasmic B23 pools and (b) increased the variability within the population. By contrast, no drastic changes were observed for B23 with AICAR.

### Impact of Phenformin, Resveratrol and AICAR on Fibrillarin

All of the compounds tested modulated the subcellular distribution of fibrillarin, albeit to different degrees (Fig. 2A). Overall, the impact on fibrillarin was small when compared to B23, nucleolin or RPA194 (Fig. 1, 3, 4). Nevertheless, significant changes occurred with phenformin (nucleus, nucleolus), resveratrol (cytoplasm, nucleolus) and AICAR (nucleolus). Furthermore, the balance between subcellular compartments was affected by all compounds. The most striking differences were uncovered by single cell analyses (Fig. 2B). Resveratrol in particular increased the heterogeneity of the population with respect to the nuclear/cytoplasmic and nucleolar/nuclear distribution of fibrillarin.

### Nucleolin is Sensitive to the Treatment with Phenformin, Resveratrol and AICAR

Nucleolin displayed the strongest response to the agents tested by us (Fig. 3). Notably, all of the compounds increased nucleolin concentrations in the nucleus and cytoplasm. In addition, phenformin and resveratrol led to a rise of nucleolin abundance in the nucleolus (Fig. 3A). Examination at the single cell level demonstrated that within the cell population all compounds led to a higher variability in the nuclear/cytoplasmic ratio, while phenformin and resveratrol also increased the heterogeneity for nucleolar/nuclear ratios (Fig. 3B). Collectively, these data suggest that phenformin, resveratrol and AICAR deregulated the nuclear/cytoplasmic steady-state localization of nucleolin.

#### RPA194 subcellular distribution is modulated by phenformin, resveratrol and AICAR

Phenformin induced small, but significant, changes for RPA194 in the nucleus and cytoplasm (Fig. 4), whereas resveratrol altered the abundance in the cytoplasm and nucleolus. On the other hand, AICAR reduced RPA194 in the nucleus and nucleolus. As a result of the changes in individual compartments, the balance nucleus/cytoplasm, nucleolus/nucleus and nucleolus/cytoplasm was significantly altered for all compounds. Interestingly, the ratios increased for phenformin and resveratrol, but were diminished for AICAR. At the single cell level, both control and treated samples displayed heterogeneity for RPA194 distribution.

Taken together, quantitative immunolocalization showed that the steady-state distribution of B23, fibrillarin, nucleolin and RPA194 is sensitive to phenformin, resveratrol and AICAR. As these proteins associate with different subcompartments of the nucleolus, our results are consistent with the idea that the compounds impinge on all major building blocks of the nucleolus. Notably, there was no uniform response to the agents, but the pattern of changes was distinct for individual proteins (summarized in Table 1).

**Table 1:** The effects of phenformin, resveratrol, AICAR or cycloheximide on nucleolar proteins are summarized.

Protein	Compound	Nuc	Cyt	Nuc/Cyt	Nucleolus	No/Nuc	No/Cyt	Nuclear retention
**B23**	phenformin	↑	↑↑	↓	↔	↔	↓	↓
	resveratrol	↑	↑	↔	↑	↔	↔	↓
	AICAR	↔	↔	↔	↔	↔	↔	↑
	CHX	↔	↔	↑	↔	↔	↔	ND
**Fibrillarin**	phenformin	↓	↔	↓	↓	↔	↓	↓
	resveratrol	↔	↔	↔	↔	↔	↔	↓
	AICAR	↔	↔	↔	↔	↔	↔	↔
	CHX	↔	↔	↔	↔	↔	↔	ND
**Nucleolin**	phenformin	**↑**	↑↑	↓	↑	↔	↓	↓
	resveratrol	**↑↑**	↑↑	↔	↑↑	↔	↔	↓↓
	AICAR	**↑**	↑↑	↔	↔	↓	↓	↔
	CHX	↔	↔	↔	↔	↔	↔	ND
**RPA194**	phenformin	↔	↔	↔	↔	↔	↔	↓
	resveratrol	↔	↓	↑	↔	↔	↓	↓
	AICAR	↔	↔	↔	↓	↔	↓	↔
	CHX	↔	↔	↔	↔	↔	↔	ND
**Nucleolar RNA synthesis**	phenformin	**↓**						
	resveratrol	**↓↓**						
	AICAR	**↓**						
**p-H3**	phenformin	**↓**						
	resveratrol	**↓**						
	AICAR	**↓**						
**DNA synthesis**	phenformin	**↓**						
	resveratrol	**↓↓**						
	AICAR	**↔**						

Results are depicted for individual compartments (nucleus, cytoplasm, nucleolus) or the distribution nucleus/cytoplasm (Nuc/Cyt), nucleolus/nucleus (No/Nuc) and nucleolus/cytoplasm (No/Cyt). The drug-induced reduction in *de novo* RNA synthesis is shown for comparison [14]. Results for control samples were defined as 1, and arrows denote the changes relative to controls: 0–0.24, ↓↓; 0.25–0.74, ↓; 0.75–1.24, ↔; 1.25–1.74, ↑; 1.75–2.24, ↑↑.

Besides DMSO and water, the vehicles for AMPK activators, we included an additional set of controls to test the impact on nucleolar protein localization. The protein synthesis inhibitor cycloheximide was selected for this purpose, because one of the downstream effects of AMPK activation is diminished translation. Although cycloheximide altered the distribution of B23 (Figure S1), fibrillarin, nucleolin and RPA194, the outcomes were clearly distinct from AMPK activators.

Collectively, our experiments support the hypothesis that AMPK activating agents have unique effects on nucleolar proteins. Importantly, the changes induced by phenformin, resveratrol or AICAR were not observed for a commonly inhibitor of translation.

### Impact of Phenformin, Resveratrol and AICAR on the Nuclear Retention of B23, Fibrillarin, Nucleolin and RPA194

**Figure 5:**
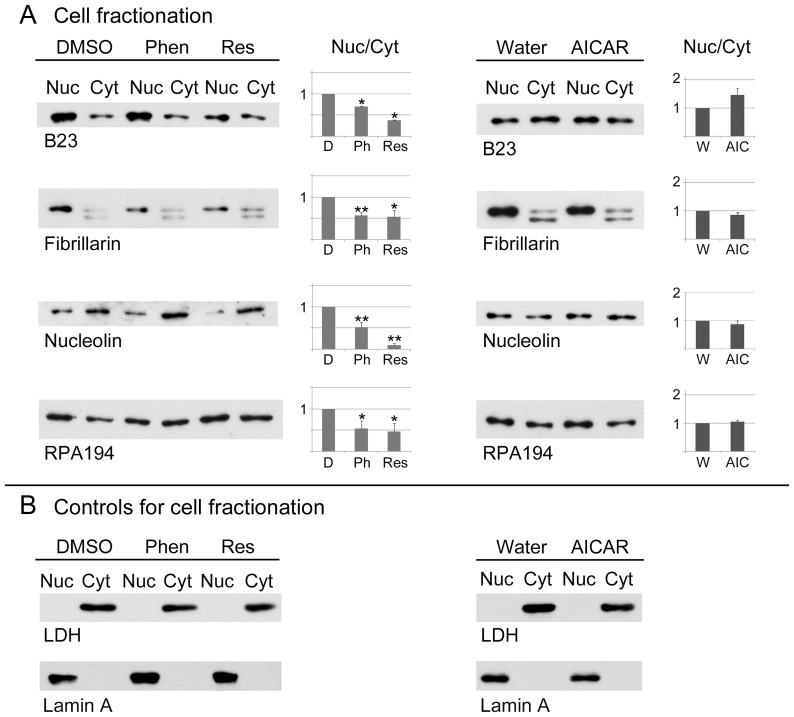
Pharmacological AMPK activators control the association of B23, fibrillarin, nucleolin and RPA194 with the nucleus. Kidney cells were incubated with vehicle (DMSO, D; Water, W), phenformin (Phen Ph), resveratrol (Res) or AICAR (AIC). Quantitative Western blotting was performed for nuclear and cytoplasmic fractions. (A) Blots were probed with antibodies against nucleolar proteins B23, fibrillarin, nucleolin or RPA194 and ECL signals were measured. The nuclear/cytoplasmic ratio was calculated; this ratio was defined as 1 for control samples. Bar graphs depict results as average+SEM for at least three independent experiments for each data point. *p<0.05; **p<0.01. (B) Control experiments evaluated the distribution of the cytoplasmic marker protein lactate dehydrogenase (LDH) and the nuclear protein lamin A.

Most cell fractionation procedures are only of limited use to determine the presence of proteins in the nucleus, because many nuclear components leak out during the preparation of nuclei [47]. However, when performed in the presence of detergent, this method is a valuable tool to examine the robustness of nuclear interactions. Under these conditions, components tightly bound to the nucleus are recovered in the “nuclear” fraction, whereas loosely bound proteins are released and co-purify with cytoplasmic constituents (Fig. 5, referred to as cytoplasm). This principle was applied previously to examine the nuclear matrix and other nuclear structures ([48] and publications citing this work). We have adapted this method here to evaluate the association of B23, fibrillarin, nucleolin and RPA194 with the nucleus (Fig. 5A) in a quantitative fashion. Specifically, nuclear/cytoplasmic (nuc/cyt) ratios were calculated, and this ratio was defined as 1 for control cells. A nuc/cyt ratio <1 indicates that in comparison to controls the protein is less firmly bound to the nucleus, whereas a ratio >1 suggest a stronger association.

Following incubation with phenformin or resveratrol, the nuc/cyt ratio was significantly smaller than 1 for all proteins. Whereas resveratrol was particularly efficient in liberating the four nucleolar proteins examined, no significant changes were observed with AICAR, although there was a slight increase for B23. For all treatments, the cytoplasmic marker lactate dehydrogenase (LDH) and the nuclear protein lamin A were restricted to cytoplasmic and nuclear fractions, respectively (Fig. 5B). Collectively, these data indicate that phenformin and resveratrol reduced the anchoring of B23, fibrillarin, nucleolin and RPA194 in the nucleus.

### Pharmacological AMPK Activators Alter the Abundance of Nucleolar Proteins

**Figure 6:**
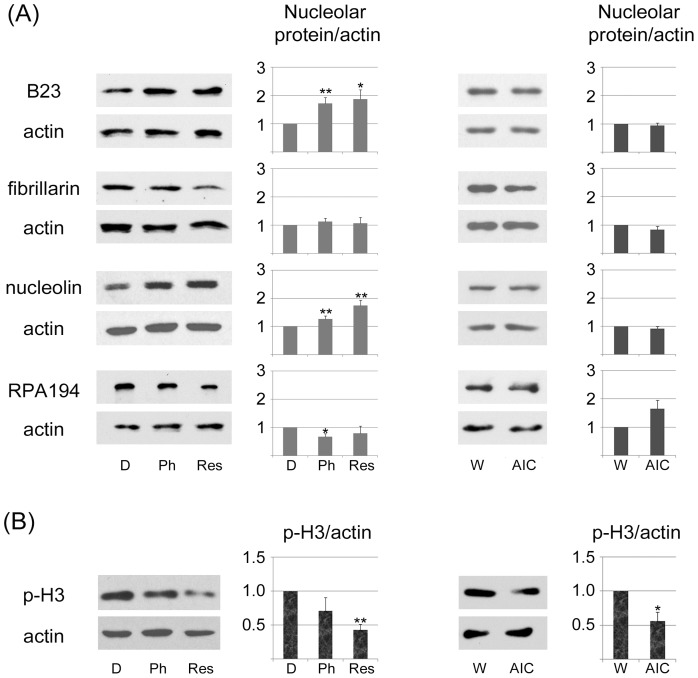
Effect of phenformin, resveratrol and AICAR on the abundance of nucleolar proteins and histone H3 phosphorylation. (A) Kidney cells were treated with vehicle or the pharmacological compound indicated. Crude extracts were probed with antibodies against B23, fibrillarin, nucleolin or RPA194; actin provided a loading control. The abundance was calculated as nucleolar protein/actin for at least three independent experiments; results were defined as 1 for control samples. Data are shown as averages+SEM; significant differences are marked with *p<0.05 or **p<0.01. (B) Signals for phospho(Ser10)-histone H3 (p-H3) were measured for crude cell extracts as described for part A.

Quantitative immunolocalization (Fig. 1–4) demonstrated that pharmacological agents modified the subcellular abundance of nucleolar proteins. This could be a consequence of relocation within the cell, either alone or in combination with changes in overall protein abundance. To obtain information on the total concentration of B23, fibrillarin, nucleolin and RPA194, crude cell extracts were prepared for control and drug-treated samples. Quantitative Western blotting (Fig. 6A) revealed that the concentration of B23 and nucleolin significantly increased with phenformin and resveratrol, whereas a small but significant reduction occurred for RPA194. By contrast, no drastic changes were observed for fibrillarin.

### Phenformin and Resveratrol Reduce de novo DNA Synthesis in Kidney Cells

As discussed above, phenformin, resveratrol and AICAR had significant impact on the organization of nucleoli. We reported earlier that each compound inhibits *de novo* RNA synthesis in the nucleolus [14]. Since nucleoli are crucial for cell growth and proliferation, it was important to further monitor the consequences of phenformin, resveratrol and AICAR on these processes. To this end, we assessed two parameters that are linked to cell proliferation, histone H3 phosphorylation on Ser10 and *de novo* DNA synthesis. H3 phosphorylation on Ser10 (Fig. 6B, p-H3), a hallmark of mitotic cells, decreased for all pharmacological agents tested, and the strongest reduction occurred with resveratrol.

**Figure 7:**
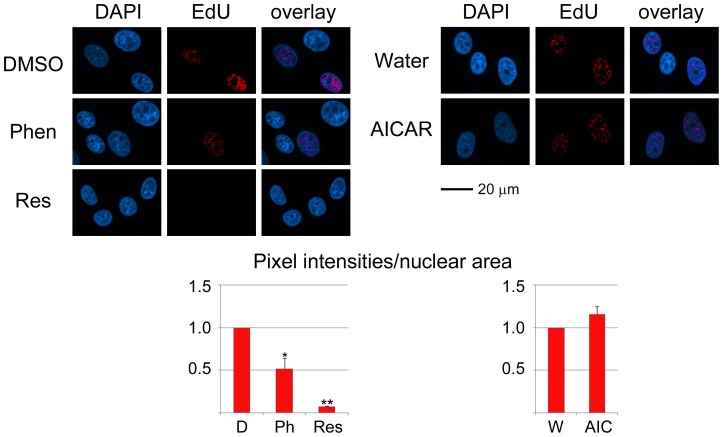
Nuclear *de novo* DNA synthesis is diminished by AMPK activators. Newly synthesized DNA was measured during the incubation with phenformin, resveratrol or AICAR. To detect DNA synthesis, EdU was added together with the pharmacological compound or vehicle. Following a 1-h incubation, cells were fixed and EdU labeled with Alexa Fluor555. Microscopic images were acquired for all samples, and nuclear pixel intensities/area were determined for at least 130 cells for each experiment and every condition. Size bar is 20 µm. Significant differences were identified with student’s *ttest*; **p<0.01; *p<0.05.

Independent experiments assessed the effect of AMPK activators on DNA synthesis. To achieve this, EdU incorporation was quantified during the treatment with phenformin, resveratrol, AICAR or vehicle. Incorporated EdU was visualized with a fluorochrome, and nuclear pixel intensities/area were measured for each condition (Fig. 7). This assay revealed a pronounced decrease in DNA synthesis for resveratrol and phenformin, while a small rise was observed with AICAR. Together, data for H3 phosphorylation and DNA synthesis support the idea that under the conditions applied here AMPK activators modulate cell proliferation.

### Short-term Incubation with Phenformin, Resveratrol or AICAR does not Trigger Apoptosis in Kidney Epithelial Cells

**Figure 8:**
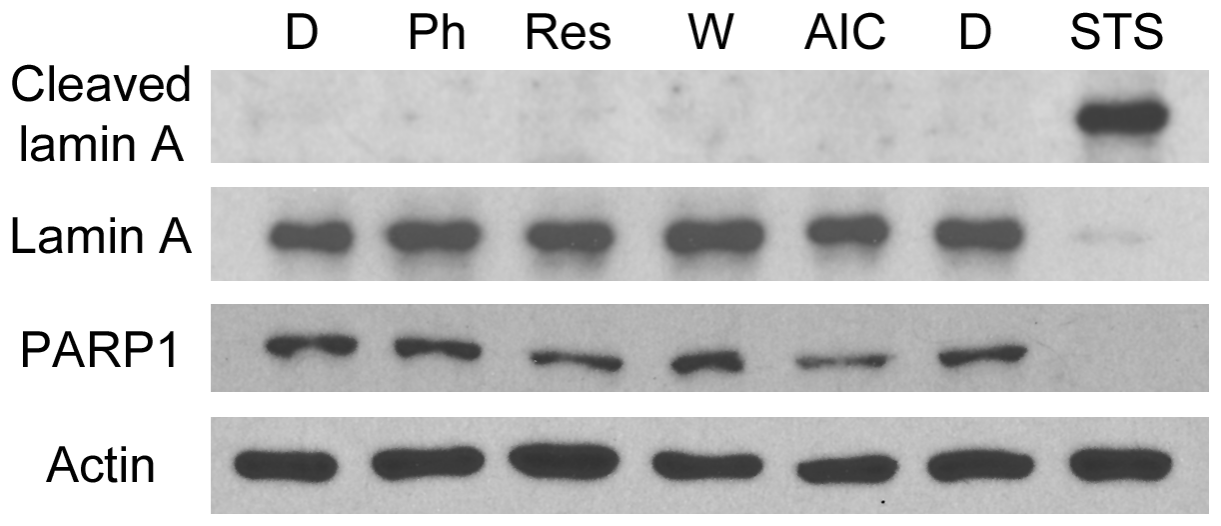
Effect of AMPK activators on lamin A and PARP1 cleavage. After 1-h treatment with vehicle (D, DMSO; W, water), phenformin, resveratrol or AICAR, kidney cells were incubated with fresh medium for 24 h. In parallel, apoptosis was induced with staurosporine (STS); the vehicle DMSO served as negative control. At the end of the 24-h incubation period, attached and floating cells were combined for each sample, and crude extracts were probed with antibodies against lamin A, PARP1 or actin. Cleavage of lamin A and PARP1, which is associated with apoptosis, occured in staurosporine-treated cells, but not in response to phenformin, resveratrol or AICAR.

**Figure 9:**
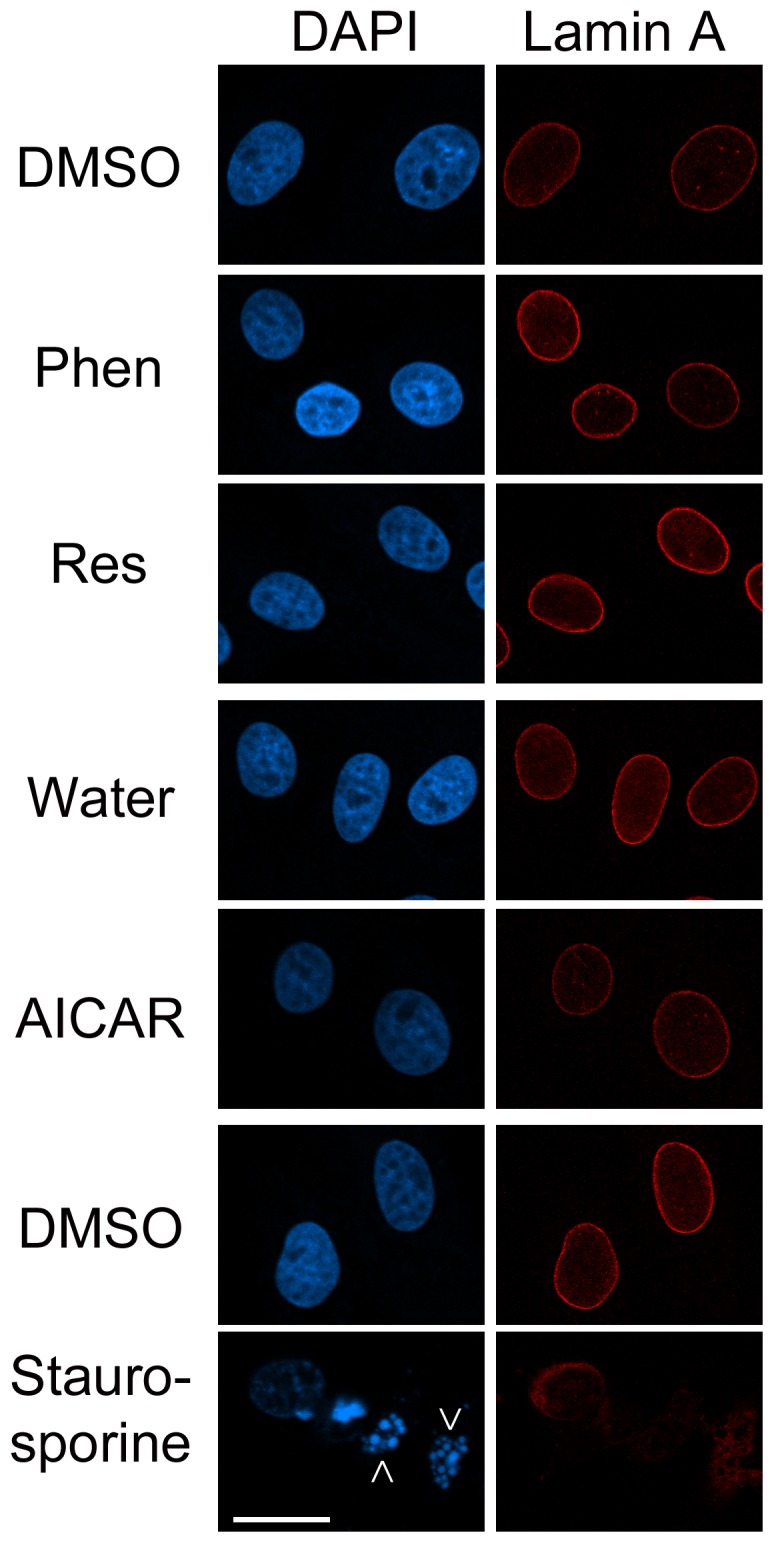
Impact of AMPK activators on nuclear integrity. Kidney cells were treated as described in Fig. 8. At th end of the 24-h incubation, cells attached to the growth support were stained with antibodies against lamin A and nuclei detected with DAPI. Fragmented nuclei are marked with arrows. Size bar is 20 µm.

AMPK activators could alter cell proliferation because they are toxic and induce apoptosis. Cleavage of lamin A and PARP1 occurs during apoptosis [49], [50], and we therefore assessed both markers. As a potent inducer of cell death in LLC-PK1 cells [41], staurosporine was included as a positive control. While staurosporine clearly led to lamin A and PARP1 cleavage, this did not take place when cells were incubated with phenformin, resveratrol or AICAR (Fig. 8). To obtain further information on drug-induced cell death, the nuclear morphology was evaluated by DAPI-staining. Nuclei were clearly fragmented upon staurosporine treatment, but not after incubation with AMPK activators (Fig. 9).

## Discussion

Nucleoli are critical for the development of kidney hypertrophy, a hallmark complication of type 1 and type 2 diabetes [11]. Yet, little is known about the changes in nucleolar biology that occur during treatment of diabetes. To begin to fill this gap, we have applied a unique experimental approach that interrogates several nucleolar proteins. To the best of our knowledge, the current study is the first to provide a quantitative in-depth analysis of nucleoli in a context that is pertinent to diabetic complications.

At the cellular level, hyperglycemia increases ribosome production in glomerular epithelial [32] and probably other kidney cells. This diabetes-induced rise in ribosome abundance is believed to promote renal hypertrophy. It is therefore conceivable that a reduction of ribosome biogenesis attenuates renal hypertrophy and the associated decline in kidney function.

Since nucleoli are the production sites of ribosomes, they provide an ideal target to develop better therapies for diabetic complications. Towards this aim, we have defined the nucleolar response to pharmacological agents by dissecting the impact on four essential nucleolar proteins.

Our previous work showed that phenformin, resveratrol and AICAR significantly decrease the first step of ribosome biogenesis, i.e. *de novo* RNA synthesis in nucleoli [14]. We demonstrate now that the experimental conditions were not toxic and did not induce apoptosis. By contrast, the treatments had significant impact on cell proliferation, as they diminished the mitotic marker phospho(Ser10)-H3. Further changes in cell proliferation were revealed for phenformin and resveratrol; both agents profoundly reduced *de novo* DNA synthesis.

By monitoring four crucial proteins that reside in different nucleolar subcompartments, we have gained a better understanding of how phenformin, resveratrol and AICAR impinge on the nucleolus. On the basis of our results, we conclude that all of these agents modulate the composition of nucleoli, as they altered the abundance of B23, fibrillarin, nucleolin and RPA194 in the nucleolus, nucleus and/or cytoplasm. Overall, phenformin and resveratrol elicited more pronounced changes when compared to AICAR. It is conceivable that these differences are linked to AMPK activity, because phenformin and resveratrol are more potent AMPK activators in our model system [14].

It is noteworthy that phenformin and resveratrol also altered the nucleus/cytoplasm balance of nucleolar proteins. Since AMPK impinges on nuclear trafficking [51], this disturbed balance could represent changes in the nucleocytoplasmic transport of B23, fibrillarin, nucleolin and RPA194. Phenformin and resveratrol not only affected the steady-state distribution of nucleolar proteins, both agents also reduced their retention in the nucleus. This loss of nuclear retention was specific and not a general property of nuclear proteins, because it was not observed for lamin A. On the basis of our results, we propose that phenformin and resveratrol release nucleolar proteins from anchors that are located in the nucleolus, nucleus or both.

The localization of all proteins analyzed here was sensitive to phenformin, resveratrol and AICAR. At the same time, B23, fibrillarin, nucleolin and RPA194 did not display a common pattern of drug-induced changes (summarized in Table 1). These differences may be explained by the distinctive drug sensitivities of the nucleolar subcompartments GC, DFC and FC; a hypothesis to be tested in the future. Although there was no common pattern, it is important that each compound affected multiple nucleolar targets. This drug-induced re-organization of nucleoli was linked to changes in nucleolar functions, as phenformin, resveratrol and AICAR inhibited RNA synthesis in the nucleolus [14].

While the present study uncovered protein-specific responses to AMPK activators, nucleolin was unique because it was significantly affected by all of the compounds tested. Although our work does not directly link cause and effect, it did reveal a striking correlation for different treatments (Table 1): the concentration of nucleolin in the nucleus was inversely related to *de novo* RNA synthesis in nucleoli [14].

Given the numerous functions of nucleolin, it is difficult to pinpoint a specific mechanism that links the protein to the changes in nucleolar organization and function, as they were elicited by pharmacological agents. Nucleolin is a multitasking protein that controls diverse cellular processes in the nucleolus and other compartments [39], [52], [53], [54], [55]. As such, nucleolin regulates rDNA transcription, pre-rRNA maturation and pre-ribosome assembly in the nucleolus. Interestingly, nucleolin can inhibit as well as stimulate rDNA transcription, and this may occur in a cell type-specific fashion [54], [55], [56], [57]. Outside of the nucleolus, nucleolin controls DNA replication, DNA repair, chromatin organization, RNA polymerase II-dependent transcription, mRNA stability and translation [54], [58], [59], [60].

In our studies, the increase of nucleolin in the *nucleus* correlated with diminished rDNA transcription. Thus, it is conceivable that nucleolin, through its localization outside of the nucleolus, inhibits the transcription of rDNA. As a possible mechanism underlying this process, nucleolin could bind and immobilize in the nucleoplasm factors which have to be present in the nucleolus for rDNA transcription. A possible candidate for such a mechanism is casein kinase 2 [61]; the enzyme is an established binding partner of nucleolin and crucial for transcription in the nucleoli [62].

One open question to be addressed in the future relates to the increase of total B23 and nucleolin concentrations upon treatment with phenformin or resveratrol. Given that both compounds reduce *de novo* RNA synthesis in the nucleolus [14] and inhibit protein synthesis through AMPK activation (reviewed in [3]), the mechanisms that promote the drug-induced rise of B23 and nucleolin levels will have to be defined. It is conceivable that the compounds alter B23 and nucleolin protein turnover, mRNA stability, transcription or mRNA translation.

In addition to identifying changes in protein abundance and overall nucleolar organization, the current study produced results at the single cell level, and thus provided new insights into the action of phenformin, resveratrol and AICAR. As individual cells varied in their response to phenformin and resveratrol, the agents generated cell populations with a heterogeneous distribution of nucleolar proteins. This variability may reflect sensitivities to phenformin and resveratrol that are cell-cycle dependent. Our results are important in the context of diabetic complications, because a more uniform cellular response may be desirable for therapeutic intervention.

Taken together, we have shed light on the cellular responses that are elicited by phenformin, resveratrol and AICAR. We demonstrate here that all of the agents alter the nucleolar organization and function. Importantly, B23, fibrillarin, nucleolin and RPA194 were identified as novel targets that are sensitive to these agents. For each target, we have quantified the drug-induced changes in (a) subcellular localization, (b) nuclear retention and (c) cellular abundance. On the basis of our data, we propose that nucleolin provides a sensitive biomarker for nucleolar functions that are pertinent to diabetic complications. Such a biomarker will improve the design of better agents that are needed to ameliorate diabetic complications. Although the action of resveratrol is promising in cell culture experiments and rodent studies of renal hypertrophy [32], this compound has low bioavailability [63], and potential drug-drug interactions during co-medication [64]. Our results suggest that the development of future drugs aimed at diabetes-induced renal hypertrophy should incorporate the nucleolus, and in particular nucleolin, as a marker to test the efficacy of new lead compounds.

## Supporting Information

Figure S1 **The response of nucleolar proteins to cycloheximide.** Kidney cells were incubated with cycloheximide as described in Materials and Methods. B23, fibrillarin, nucleolin and RPA194 were examined in control and treated samples as described for Fig. 1. Size bar is 20 µm. Note that nucleolar proteins react differently to cycloheximide and AMPK activating agents.
(TIF)
