# Supplementary figures and images for: Pharmacological AMP Kinase Activators Target the Nucleolar Organization and Control Cell Proliferation
Source: PLoS One. 2014 Jan 30;9(1):e88087. doi: 10.1371/journal.pone.0088087 (PMC3907577; doi:10.1371/journal.pone.0088087)

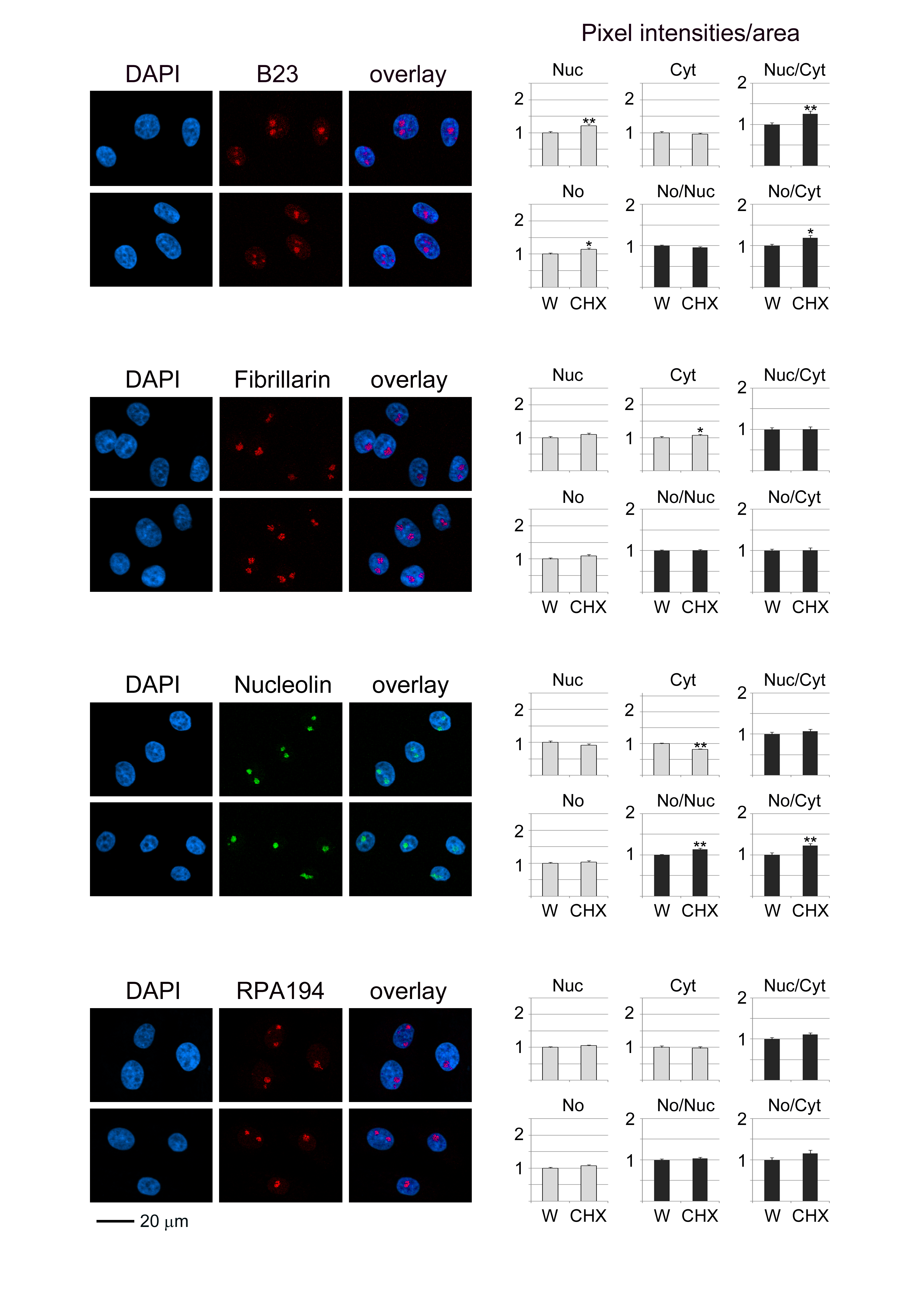

Supplement: Figure S1 — The response of nucleolar proteins to cycloheximide. Kidney cells were incubated with cycloheximide as described in Materials and Methods. B23, fibrillarin, nucleolin and RPA194 were examined in control and treated samples as described for Fig. 1. Size bar is 20 µm. Note that nucleolar proteins react differently to cycloheximide and AMPK activating agents. (TIF) [file pone.0088087.s001.tif]
